# Reflections on workplace adjustments for pregnant employees: a qualitative study of the experiences of pregnant employees and their managers

**DOI:** 10.1186/s12884-022-04749-1

**Published:** 2022-06-01

**Authors:** Dorte Raaby Andersen, Anne-Mette Hedeager Momsen, Pernille Pedersen, Rikke Damkjær Maimburg

**Affiliations:** 1 Department of Occupational Medicine, University Research Clinic, Danish Ramazzini Centre, Goedstrup Hospital, Herning, Denmark; 2 DEFACTUM, Central Denmark Region, Marselisborg Center, Aarhus, Denmark; 3 Department of Public Health, Aarhus University, Aarhus, Denmark; 4 Department of Clinical Medicine, Aarhus University, Aarhus, Denmark; 5 Department of Gynaecology and Obstetrics, Aarhus University Hospital, Aarhus, Denmark; 6 School of Nursing and Midwifery, Western Sydney University, Sydney, Australia

**Keywords:** Pregnant employees,, Workplace adjustments,, Work environment,, Workplace intervention

## Abstract

**Background:**

The European Union directive requires employers to assess and ensure safety measures for pregnant women in the workplace. Despite this, the rate of sick leave among pregnant Scandinavian women is relatively high. This study aims to provide insight into how pregnant employees and their managers experience and address pregnancy at the workplace, to identify preconditions for successful workplace adjustments for pregnant women.

**Methods:**

We carried out a qualitative study that involved semi-structured interviews with seventeen participants: eight pregnant women and nine managers from occupations whose employees demonstrate an increased likelihood of taking sick leave during pregnancy. The interviews were thematically coded and organized into main themes and subthemes.

**Results:**

Based on semi-structured interviews with the seventeen participants (eight pregnant employees and nine managers), we identified preconditions for successful workplace adjustments. According to the pregnant employees, these included, “The managers' concern, understanding, and acknowledgment,” “support and acceptance from colleagues,” and “pregnant employees’ acceptance of their need for adjustments.” According to the managers, the preconditions for successful workplace adjustments included “an open and honest dialogue” and “a systematic approach.”

**Conclusion:**

Implementing workplace adjustments for pregnant employees is a complex process that comprises various initiatives, and their success may depend on several factors. This study’s findings suggest that the success of workplace interventions depends on 1) management, colleagues, and the pregnant employee recognizing and accepting pregnant women’s needs, 2) an organizational culture that supports women and pregnancy without compromising the occupational health of other employees, and 3) professional guidance that supports both women and managers when dealing with pregnancy-related concerns. We suggest that this study’s findings may be used to improve the implementation of workplace adjustments for pregnant women.

## Background

In Scandinavia, it is common for women to work during pregnancy. The European Union directive requires employers to assess safety measures to ensure the health of pregnant women at the workplace [1]. Despite this, the rate of sick leave among pregnant Scandinavian women is relatively high [2, 3]. Two-thirds of pregnant women take 48 to 73 days of sick leave while pregnant [3–6]. Sick leave has been associated with individual factors such as maternal age, being overweight, socioeconomic status, and assisted reproductive therapy [5, 6]. Physical work factors (e.g., heavy lifting, and night or shift work) and stressful working conditions have also been identified as factors that increase the likelihood of sick leave during pregnancy [7]. Workplace adjustments have been found to reduce the number of days of sick leave taken by pregnant women. However, studies indicate that not all pregnant women who need such adjustments find that they are made [8–12]. A review by Salihu et al. (2012) recommended that occupational risk assessments be conducted for pregnant employees [13]. They also suggested that the assessments may be made by the employee’s healthcare provider, as well as by occupational medicine clinics [13]. This is especially important for women exposed to radiation, solvents, and chemicals at their workplaces. Moreover, Salihu et al. (2012) emphasize that even though manual labor and physically demanding jobs do not pose major risks to fetal health, a combination of significant physicality and other factors, such as long work hours, should be reduced, as a moderate, temporary reduction in physical exertion may improve maternal and fetal health. Last, they note the importance of giving attention to women with a history of pregnancy-related complications, and to the workplace culture [13].

There is limited evidence concerning interventions that target sick days among pregnant employees in healthcare settings, and only a few studies have been conducted at workplaces [14]. In Denmark, a randomized trial was conducted to evaluate whether educating managers reduced absenteeism among pregnant employees [15]. Managers were taught about occupational risk factors related to pregnancy, and instructed to make workplace adjustments systematically and on a case-by-case basis. This study demonstrated that providing managers with a short educational program was insufficient to reduce absenteeism in hospital and day-care settings during pregnancy [15]. Training programs may increase managers' knowledge; however, they have not proven sufficient to reduce sick days. Implementing workplace adjustments is a complex process that comprises various initiatives, and their success may depend on several factors. To understand the complex challenges faced by pregnant employees and their managers, and to understand how to support both groups when implementing workplace adjustments, we conducted a qualitative study of pregnant employees and their managers, in which we:

• explored how pregnant employees and their managers experience and manage pregnancy at the workplace,
• identified preconditions for successful workplace adjustments for pregnant women, based on pregnant employees’ and managers’ experiences.

The information we gathered may help improve the implementation of workplace adjustments in sectors that present an increased likelihood of sick leave. This is relevant to interventions that target sick days taken by pregnant employees.

## Methods

This was a qualitative study that involved semi-structured interviews with pregnant employees and their immediate managers. The interviews aimed to explore how pregnant employees and their managers experienced and managed pregnancy at the workplace, and based on this, to identify preconditions for successfully implementing workplace adjustments for pregnant women.

### Study participants

We wanted to explore how pregnancy is experienced by women working in sectors with an increased likelihood of pregnancy-related sick leave. In Denmark, pregnant employees in restaurants and hotels, healthcare, travel agencies, cleaning jobs, and retail reportedly took sick leave two to three times more often than female employees who were not pregnant in these sectors [16]. We selected a purposive sample to find participants who were employed in these fields. The goal was to include participants with diverse experiences, and to gain in-depth knowledge. The pregnant women were recruited from respondents to a survey carried out at six prenatal midwife clinics in the Central Denmark Region, from March to October 2018. The inclusion criteria for managers were at least five years of management experience with pregnant employees, and responsibility for at least thirty employees. The pregnant employees were invited by telephone after they had agreed to be contacted, and the managers were recruited by contacting their workplaces. We stopped adding participants when we reached data saturation and the interviews contributed little or no new information.

We conducted semi-structured interviews. The topics in the interview guides (one for the employees and one for the managers) were inspired by existing literature that showed that work-related factors that increased the likelihood of sick leave included psychosocial work factors (e.g., support from supervisors and colleagues) and physical work factors (e.g., heavy lifting, night or shift work, standing work). The literature also inspired us to include questions about the attitude to sick days [17]. The topics in the interview guide were: “Pregnancy at the workplace,” “Attitudes to sick leave during pregnancy,” “Workplace adjustments,” “Relationship to the manager,” “Relationship to colleagues,” “Role of physicians and midwives,” and “The possibility of, and barriers to, keeping pregnant employees on the job throughout pregnancy.” We attempted to balance a focused exploration of the topics in the interview guide and an open approach. Some of the subtopics that emerged during the first interviews were included in subsequent interviews (e.g., descriptions of specific challenges).

### Data collection

The interviews lasted between 30 and 120 min, and were conducted in settings chosen by the participants. We interviewed the managers face-to-face and at their workplaces. Except for two interviews with pregnant women that took place in the first author's office, the interviews were conducted over the telephone, at the participants’ request. We requested the participants’ permission to record the interviews, and all participants provided informed consent. Having consented, participants were assured that the information provided would be treated as confidential, and that their participation would be anonymous. All interviews were transcribed verbatim. The interviews were conducted in Danish and have been translated into English.

### Data analysis

**Fig. 1:**
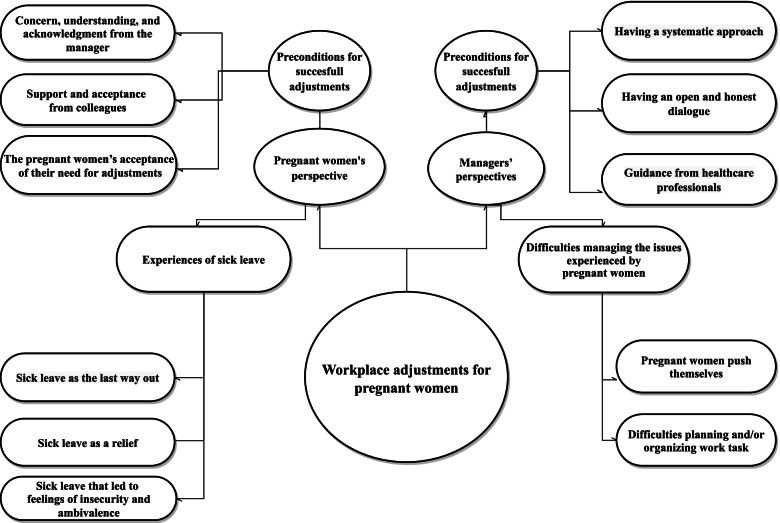
"Thematic mapping of the experiences of pregnant women and their managers "

A constructionist perspective (meaning and experience are socially produced and reproduced, rather than inherent) underpinned our thematic analysis [18], which explored the experiences of pregnant employees and their managers, and based on this, we identified preconditions for successful workplace adjustments. The analysis was inspired by a deductive approach, as some of the pre-established topics were expected to be reflected in the data. We began by carefully reading the interviews. We then coded the interviews thematically, using the qualitative software program, NVIVO 11. We coded everything considered relevant or potentially interesting. Then, we went through the coded passages and tried to identify similar phrases and patterns, as well as differences in the materials. We grouped the codes into themes that were broader than the codes. We reflected on the purpose of this study, and went through the codes and decided to discard those that were too vague or not relevant enough (interview themes that were considered less relevant to a workplace context were excluded from further analysis). After reflecting on the themes, we returned to the data. We looked for nuances and differences in the experiences of the pregnant employees and their managers, and both positive and negative experiences of pregnancy at the workplace. In this process, we developed subthemes and merged some of the themes. With a finalized list of themes, we revisited each theme and renamed some of them for conciseness and clarity, and to ensure that various voices were represented in the subthemes (For an overview of the thematic mapping, see Fig. 1).

## Results

Based on semi-structured interviews with seventeen participants—eight pregnant employees and nine managers—we explored how pregnant employees and their managers experience and manage pregnancy at the workplace. Based on the pregnant employees’ descriptions of being pregnant at work, we identified the themes, “Preconditions for successful workplace adjustments” and “Pregnant women’s experiences of sick leave.” Based on the managers’ descriptions of having a pregnant employee, we identified the themes, “Preconditions for successful workplace adjustments” and “Difficulties of managing the problems experienced by pregnant employees.” For each theme, we organized the descriptions into sub-themes. Each sub-theme illustrates nuances and differences in the experiences of the pregnant employees and their managers. In the following sections, we present each of the main themes and the sub-themes in detail.

### Pregnant employees’ perspective: preconditions for successful workplace adjustments

Although most of the pregnant women in this study continued to work while pregnant, work was not always easy. Pregnant women may experience various pregnancy-related symptoms that may present a challenge in the workplace, and that need to be resolved, if she is to continue to work during her pregnancy. The pregnant women in this study experienced pregnancy-related symptoms such as pelvic pain, fatigue, and Braxton-Hicks contractions. Several of the pregnant women explained that they and their managers collaborated in an effort to meet these challenges by implementing workplace adjustments, such as flexible schedules, avoiding certain tasks, and reducing work hours. However, the success of these workplace adjustments varied. Based on the pregnant employees’ descriptions, we identified three preconditions for positive experiences of workplace adjustments. These include: “concern, understanding, and acknowledgment from the manager,” “support and acceptance from colleagues,” and “the pregnant women’s acceptance of their need for adjustments.” In the following sections, we present each of these sub-themes in detail.

#### The managers’ concern, understanding, and acknowledgment

The pregnant employees’ descriptions of successful adjustments to their work during their pregnancy included instances where their managers showed concern, understanding, and acknowledgment of their needs. Some of the pregnant employees explained that their manager was very understanding, and helped them with information about handling pregnancy at work. Several women also mentioned that they had extensive contact with their managers while pregnant, and that their managers helped them implement workplace adjustments (Pregnant employee 4). This acknowledgement from the managers, together with their paying attention and listening to their pregnant employees, seemed to be an important precondition for successful workplace adjustments.

In contrast, pregnant employees’ descriptions of unsuccessful efforts to implement workplace adjustments included descriptions of managers who did not pay attention, did not take the initiative when talking to their pregnant employees, or did not support or respect the agreements on workplace adjustments. One woman explained that she felt like a burden to her former employer, who did not respect her needs. Her manager had promised to reduce her work hours, but she still felt she had to work more than he had promised. She explained: “They [The managers] did not argue for sick leave, and when I had to go on part-time sick leave, I was still working 37 hours a week” (Pregnant employee 6). Another example of a negative experience with a manager was a manager who had previously ignored a pregnant employee’s request for adjustments at work, and therefore she continued to conceal her pain. She did not communicate much with her manager during her pregnancy, and she did not expect the manager to listen her. She did not even bother to ask her what to do about her pregnancy-related challenges. “I wouldn’t even waste my time on that,” she reported (Pregnant employee 1), and explained how her unpleasant work conditions and her need for adjustments had been ignored by her manager several times. The manager’s lack of concern, understanding, and acknowledgment prevented this pregnant employee from having a positive experience of workplace adjustments.

#### Support and acceptance from colleagues

The pregnant employees’ descriptions of positive experiences of workplace adjustments also mentioned colleagues’ support and acceptance. One pregnant employee explained that her colleagues helped reorganize the work to relieve her as much as possible, and she emphasized that her colleagues paid significant attention to her condition, which she appreciated: “Actually, it was very nice that somebody said, ‘now, we are a bit worried about you’” (Pregnant employee 5). These expressions of colleagues’ support and acceptance indicate that the pregnant employees did not necessarily feel entitled to recognition of pregnancy-related needs. Colleagues’ supportive attitude seemed to be an important precondition for successful workplace adjustments.

In contrast, pregnant employees' negative experiences of workplace adjustments included colleagues’ failure to recognize their needs. The pregnant employees described this as very painful. According to one woman, the worst aspect of workplace adjustments during her pregnancy was the lack of acknowledgment from a group of colleagues. She felt that they did not understand, and that they disapproved of the adjustments because such adjustments had not been made when they were pregnant. One woman felt that they did not understand her needs and thought the adjustments unfair. She explained:

They thought, “Why does she get half an hour? We didn't get that when we were pregnant!” That, I would say, was the hardest thing for me. When they were pregnant, those things weren’t considered. (Pregnant employee 8)

As the foregoing statement illustrates, colleagues’ lack of support and understanding seems to form a great barrier to positive experiences of workplace adjustments for pregnant employees.

#### Pregnant employees’ acceptance of their need for adjustments

The pregnant employees’ descriptions of positive experiences of workplace adjustments also included descriptions of their own acceptance of their need for adjustments. One woman explained that she thought it was easier to take care of herself now that she was pregnant. She explained: “I became a little calmer, and took a bit more care of myself when I got pregnant” (Pregnant employee 3). She accepted her pregnancy-related needs, and thought that her pregnancy was a legitimate reason to take better care of herself. In contrast, other pregnant employees described their difficulty in accepting their need for workplace adjustments, and their problems with accepting the strain they felt while pregnant. Some disliked being dependent on help from their colleagues. One woman mentioned that adjustments had been made for her, but it was difficult for her to take advantage of these adjustments, because she perceived herself as hardworking and tenacious. She refused to be ﻿“a burden to her colleagues﻿”, and although they accepted the adjustments, she could not accept them (Pregnant employee 6). The pregnant employees’ acceptance of their need for adjustments seemed to be an important precondition for successful workplace adjustments. Failure to accept their needs seemed to be a barrier to successful workplace adjustments.

In this study, the pregnant employees’ descriptions of their positive and negative experiences of workplace adjustments for pregnancy revealed that acknowledgment, acceptance, and approval of these adjustments by the manager, colleagues, and the woman herself are important to employees having a positive experience of being pregnant at the workplace. All these aspects seem to be preconditions for successful workplace adjustments for pregnant women.

### Pregnant employees’ perspective: experiences of sick leave

Some of the employees in this study were more challenged by their pregnancies than others, and some had to take sick leave, whereas others continued to work until their scheduled maternity leave. Based on the pregnant employees’ descriptions, we identified three different experiences of sick leave: “sick leave as the last option,” “sick leave as a relief,” and “sick leave that led to feelings of insecurity and ambivalence.”

#### Sick leave as the last option

Most of the pregnant women in our study expressed a wish to remain active at their workplace for as long as possible while pregnant. They described the importance of maintaining their relationships with their colleagues, and they wanted to continue working, as work was important to them. One woman reported that she had pressured herself, and continued to work for too long, because she wanted to work throughout her pregnancy. Another woman reported that she wanted to remain on the job, even though her manager suggested sick leave. She explained:

I have been my own worst enemy, because I have had difficulty saying “no” at my workplace. I like my job, and I don’t want to be at home, so it’s been a difficult balance. […] In fact, my manager would have preferred that I called in sick right away, to take care of myself. But I did not [...] Looking back, I should have done it [taken sick leave] long before. But it is hard when you're happy with your work, you're happy with your workplace. (Pregnant employee 7)

This woman was not the only one who talked about sick leave as "the last option." Other women expressed this in terms of "making it just until maternity leave," which also indicates the employees’ strong desire to remain on the job throughout their pregnancy, despite the pregnancy-related challenges they faced.

#### Sick leave as a relief

Some women found sick leave a relief, because it allowed them to take care of themselves. One woman explained that taking sick leave was a positive thing. She explained, “It was a relief that now I could do things at my speed” (Pregnant employee 1). Similarly, another woman reported that her manager’s suggestion of a period of sick leave left her with a feeling of “relief,” but also with a feeling of ambivalence. She described it in these terms:

Well, it [the suggestion to take sick leave] was both positive and negative. On the one hand, it was somehow a relief. Well, then he [the manager] said it was okay that I am sick and that I must take care of myself. On the other hand, it was also kind of, “well but I'm not doing my job well enough” and “what's the reason?” (Pregnant employee 2)

Pregnant employees’ descriptions of taking sick leave reveal that this may give rise to various feelings. The suggestion of sick leave may conflict with some women’s strong desire to continue working throughout their pregnancy, but other women may find it a relief to be able to fully take care of themselves not worrying about their job. Lastly, the descriptions indicate that suggesting sick leave may lead to feelings of insecurity and ambivalence when the pregnant woman is unsure why the manager is suggesting this.

### Managers’ perspectives: “preconditions for successful workplace adjustments”

The managers’ experiences with pregnant employees varied. They found that some of their pregnant employees made several adjustments and took sick leave while pregnant, whereas others remained on the job. Based on the managers’ descriptions of having a pregnant employee, we identified the following preconditions for successful workplace adjustments: “an open and honest dialogue” and “a systematic approach.” We also identified the following barriers to successful workplace adjustments, from the perspective of the managers: “pregnant employees push themselves” and “difficulty planning and/or organizing work-related tasks.” In the following sections, we present each of the main categories and the subcategories in detail.

#### An open and honest dialogue

The managers emphasized that an open and honest dialogue with the employees was a prerequisite for addressing pregnant employees’ needs in the workplace. Some managers reported that they depended on pregnant employees being open and honest about their need for adjustments (between workplace-adjustment meetings), as the managers did not always have time for individual follow-up. One manager emphasized the need for open dialogue as follows:

[…] I cannot see it or feel it (the need of the pregnant woman) […] Be open and honest about it, and come to us, because then maybe we can adjust the work. (Manager at a hotel)

The managers explained that if pregnant employees did not talk to them, it was difficult for them to act in time. For instance, one manager talked about her experiences with two pregnant employees, where one was open and honest, which made it possible to adjust her work, whereas another woman suddenly said she was unable to work. The manager was surprised, because she did not know that the latter pregnant employee had suffered as a consequence of working during her pregnancy. She explained:

We had one pregnant employee who managed to remain on the job until six weeks before she gave birth. She was working and doing her best. And she was really good at letting me know, “I can feel in my body that it is hard to wash the floor.” We communicated well, and it worked really well. But on the other hand, there was another pregnant employee who, after three months of pregnancy, said that she could not continue working, and that her doctor agreed with her on that. (Manager at a cleaning company)

These statements from managers indicate that honest dialogue is a prerequisite for addressing pregnant employees needs in the workplace, and a precondition for successful workplace adjustments that may help pregnant women to continue work.

#### A systematic approach

The managers’ descriptions of positive experiences of workplace adjustments included experiences of working systematically when implementing workplace adjustments for pregnant employees. They explained that they followed their workplace’s pregnancy policy, and used workplace assessments when they had a pregnant employee. They called the pregnant woman in for a meeting, and followed up on her situation throughout her pregnancy. In this way, they were able to adjust work-related tasks if needed. Most of the managers worked very systematically. The one manager who did not work as systematically as the others considered changing her informal practices, because of the advantages of doing so. When she considered the matter, she thought it would be easier for her to remember to talk to the pregnant woman if it was a part of a formal procedure. Furthermore, she thought it would be easier for the pregnant woman to come to her.

### Managers’ perspectives: difficulties of managing the problems experienced by pregnant employees

#### Pregnant employees push themselves

The managers sometimes had difficulty managing the problems experienced by pregnant employees. Several managers mentioned that some pregnant employees pushed themselves too hard. In the interviews, they commented on the question of pregnant employees who they thought were too proud to ask for and accept workplace adjustments. One of the managers described an employee who was usually capable of lifting as much as the male employees in the shop, but during her pregnancy she was not able to work and do the heavy lifting as she was used to. It was difficult for her to adjust to her new situation, and therefore the manager had to be aware of this, and keep telling her to take care of herself, instead of pushing herself too hard. When pregnant employees had difficulty accepting their need for workplace adjustments, the managers felt that they had to be more attentive to their difficulties.

#### Difficulties planning and/or organizing work-related tasks

Managers mentioned challenges related to planning and organizing work-related tasks, because the pregnant employees’ condition sometimes changed from one day to the next, and they would lack the time to make useful, alternative arrangements. Moreover, they did not always know what to expect, or how much to demand of pregnant employees. Therefore, some of them asked pregnant employees about their healthcare providers’ recommendations. These recommendations were often used to justify and legitimize workplace adjustments or absences from the workplace, and they sometimes made it easier for pregnant employees and their managers to balance the workplace adjustments, and informed them about how to respond to various symptoms. Healthcare providers seemed to play a key role in offering advice about a given situation, and when consulted, they sometimes had a strong influence on future adjustments and developments in pregnant employees’ work. According to the managers, pregnant employees sometimes managed to remain on the job because of guidance from these professionals; in other situations, they immediately recommended sick leave, because of the job’s demands.

Finally, managers mentioned possible challenges and dilemmas related to the fact that adjustments for pregnant employees should not adversely affect other employees. One manager emphasized that “It must not be that pregnant employees are penalized […] It is a balance” (Manager, hotel).

These statements by managers reveal a possible challenge, as they are responsible for managing the workplace and ensuring workplace adjustments for pregnant employees, and at the same time, they must balance the probability of an increased workload for the other employees, which is often uncompensated.

## Discussion

This study was based on interviews with pregnant employees and their managers, which provided insight into how these groups experience pregnancy in the workplace, and the preconditions for successful workplace adjustments for pregnant women.

Overall, the statements by women and managers about work and pregnancy revealed different experiences of implementing workplace adjustments for pregnant women. Workplace adjustments were difficult to accept for some pregnant employees, who worried about how they would be perceived by others, and how it would affect their self-image and social identity. This concern with self-presentation is consistent with the findings of other studies that indicate that pregnant employees have strong professional identities [19, 20], and supports social identity theory with respect to how people try to live up to their self-image and social identity. In this study, findings regarding pregnant employees’ concerns about being judged negatively by their colleagues or managers were similar to results reported by Severinsen et al. (2018), who also found indications that pregnant employees try to live up to their own expectations, as well as to societal and workplace norms [20]. Pregnant employees seemed to depend on acceptance and support from both management and their colleagues. This suggests that although an organization or workplace may have policies for workplace adjustments during pregnancy, colleagues’ acceptance is essential to a positive experience of being pregnant at the workplace. If the workplace culture suggests that pregnant employees will be regarded differently if they accept these adjustments, then it may be difficult for them to do so. This is consistent with an American study that showed that the fear of confirming stereotypes of pregnant employees' work attitudes may drive pregnant employees to work even harder than usual, and thereby risk injury or complications [21].

Our study showed that colleagues’ and management’s acceptance and recognition of pregnant employees needs seemed to be important preconditions for successfully implementing workplace adjustments for pregnant women.

Managers’ descriptions of addressing pregnancy-related concerns illustrate how they must balance the needs of pregnant employees and the daily operation of the workplace. Even though the managers mentioned the possibility of making workplace adjustments, it seemed that contextual factors, such as general workload, difficulties related to planning and organizing tasks, and colleagues’ acceptance, played a part in how those adjustments were implemented. This reveals a possible tension or dilemma related to addressing pregnant employees’ needs for job adjustments and their co-workers’ needs. In an interview study with pregnant employees, these women requested alternative uses of their skills, so they could remain on the job throughout their pregnancy [20]. The lack of time (to find relevant and/or alternative solutions) mentioned by some of the managers who participated in this study underscores their dilemma when it comes to balancing the efficiency of managing the workplace and workplace adjustments for pregnant employees without compromising the occupational health of the other employees. Further research is needed to identify the potential of more creative workplace adjustments, and to explore possible solutions to this dilemma. This may also involve the executive management level.

In this study, guidance from healthcare providers also played a significant role. Statements from healthcare providers were used by pregnant employees to legitimize pregnancy-related adjustments, and sometimes helped pregnant employees and managers to deal with pregnancy-related issues and workplace adjustments. Guidance from healthcare providers in addressing pregnant workers was also appreciated and sometimes requested by the managers. However, Backhausen et al. (2021) found that an important reason for inadequate adjustments of work-related tasks and the retention of pregnant employees was a lack of communication with general practitioners or midwives, who recommend sick leave, leaving no means to find a solution to remain at work [12]. The role of health professionals and ways to strengthen communication between healthcare providers and the workplace may be relevant to explore further.

### Strengths and limitations of this study

This study provides detailed information on the complexity of addressing pregnancy in the workplace from the perspective of pregnant employees and their managers. It provides not only insight into preconditions for successful workplace adjustments but also the complex challenges and barriers faced by pregnant employees and their managers.

However, this study has some limitations. The first limitation is the small number of participants. Even though we did not stop including participants until we reached saturation, this study may have benefited from including more participants from each of the sectors. This would have enabled us to provide an analysis of the experiences of managers and pregnant employees between sectors. Furthermore, it was not possible to include participants based on sick leave, age, or other background variables. If so, it would have been possible to examine patterns and would have enabled a comparative analysis between groups. It has, however, been possible to identify patterns in the statements of the pregnant employees and managers, despite the number of participants, which contributes to valuable knowledge in this field.

## Conclusions

Implementing workplace adjustments for pregnant employees is a complex process that comprises various initiatives, and their success may depend on several factors. This study’s findings suggest that the success of workplace interventions depends on 1) management, colleagues, and the pregnant employee accepting and recognizing pregnant women’s needs, 2) an organizational culture that supports women and pregnancy without compromising the occupational health of other employees, and 3) professional guidance that may support both women and managers when dealing with pregnancy-related concerns. We suggest that this study’s findings may be used to improve the implementation of workplace adjustments for pregnant women.

## Data Availability

The datasets generated and/or analysed during the current study are not publicly available due to ethical reasons but are available from the corresponding author on reasonable request.
