# Correction: Reflections on workplace adjustments for pregnant employees: a qualitative study of the experiences of pregnant employees and their managers

**DOI:** 10.1186/s12884-022-05333-3

**Published:** 2023-01-03

**Authors:** Dorte Raaby Andersen, Anne-Mette Hedeager Momsen, Pernille Pedersen, Rikke Damkjær Maimburg

**Affiliations:** 1 Department of Occupational Medicine, University Research Clinic, Danish Ramazzini Centre, Goedstrup Hospital, Herning, Denmark; 2 DEFACTUM, Central Denmark Region, Marselisborg Center, Aarhus, Denmark; 3 Department of Public Health, Aarhus University, Aarhus, Denmark; 4 Department of Clinical Medicine, Aarhus University, Aarhus, Denmark; 5 Department of Gynaecology and Obstetrics, Aarhus University Hospital, Aarhus, Denmark; 6 School of Nursing and Midwifery, Western Sydney University, Sydney, Australia

**Correction: BMC Pregnancy Childbirth 22, 456 (2022)**

**https://doi.org/10.1186/s12884-022-04749-1**

Following publication of the original article [1], the authors identified an error in the author name of Rikke Damkjær Maimburg.

The incorrect author name is: GivenName: Rikke, FamilyName: Damkjær Maimburg

The correct author name is: GivenName: Rikke Damkjær, FamilyName: Maimburg

The author group has been updated above and the original article [1] has been corrected.
